# Supplementary material for: Naturally occurring fire coral clones demonstrate a genetic and environmental basis of microbiome composition
Source: Nat Commun. 2021 Nov 4;12:6402. doi: 10.1038/s41467-021-26543-x (PMC8568919; doi:10.1038/s41467-021-26543-x)
Supplement: Supplementary file 1 — Supplementary information [file 41467_2021_26543_MOESM1_ESM.pdf]

Supplementary Information

Naturally occurring fire coral clones demonstrate a genetic and environmental basis of microbiome composition

Dubé CE, Ziegler M, Mercière A, Boissin E, Planes S, Bourmaud CAF, Voolstra CR

Contents

Supplementary Table 1. Two- and one-way permutational multivariate analysis of variance (PERMANOVA) showing the effect and interaction of 'Genotype' and 'Habitat' on bacterial ASV composition (two-way analysis represents the overall variation and one-way analysis represents genetic and environmental variation, separately).

Supplementary Table 2. One-way permutational multivariate analysis of variance (PERMANOVA) showing the effect of 'Colony size' on bacterial ASV composition.

Supplementary Figure 1. Temperature (**A**) and light (**B**) profiles measured at each of the habitats over time.

Supplementary Figure 2. LEfSe analysis displaying predicted functional profiles of the habitat-specific bacterial ASVs for MetaCyc pathways (**A**) and levels 1–3 for individual KOs (**B**).

Supplementary Table

Supplementary Table 1. Two- and one-way permutational multivariate analysis of variance (PERMANOVA) showing the effect and interaction of 'Genotype' and 'Habitat' on bacterial ASV composition (two-way analysis represents the overall variation and one-way analysis represents genetic and environmental variation, separately).

Factor per analysis	*df*	*F*	*R2*	*p*
**Overall variation (Two-way)**				
Genotype	5	1.9494	0.0678	0.0001
Habitat	*2*	2.8612	0.0398	0.0001
Genotype X Habitat	7	1.1788	0.0574	0.0024
**Genetic variation (One-way and Post-hoc test)**				
Mid Slope	3	1.2255	0.1432	0.042
MD_G2 and MD_G6	1	1.4013	0.0910	0.020
Upper Slope	5	1.8340	0.0984	0.0001
UP_G1 and UP_G3	1	1.3465	0.0381	0.043
UP_G1 and UP_G4	1	2.3949	0.0403	0.001
UP_G1 and UP_G5	1	1.8782	0.0471	0.001
UP_G1 and UP_G6	1	2.5177	0.0530	0.001
UP_G2 and UP_G3	1	1.3646	0.1852	0.032
UP_G2 and UP_G4	1	1.5121	0.0496	0.006
UP_G2 and UP_G5	1	1.3187	0.1165	0.023
UP_G2 and UP_G6	1	1.4811	0.0801	0.003
UP_G3 and UP_G4	1	1.3745	0.0452	0.018
UP_G3 and UP_G5	1	1.5943	0.1375	0.003
UP_G3 and UP_G6	1	1.7262	0.0922	0.002
UP_G4 and UP_G5	1	1.6254	0.0469	0.001
UP_G4 and UP_G6	1	2.4165	0.0570	0.001
UP_G5 and UP_G6	1	2.0292	0.0469	0.001
Back Reef	4	1.1111	0.2410	0.072
**Environmental variation (One-way)**				
G1	1	1.8359	0.0950	0.0001
G2	1	1.3700	0.1741	0.0062
G3	1	1.3509	0.1838	0.0338
G4	1	1.3700	0.1741	0.0049
G5	1	2.0273	0.1556	0.0012
G6	1	1.4294	0.0700	0.0035

Supplementary Table 2. One-way permutational multivariate analysis of variance (PERMANOVA) showing the effect of 'Colony size' on bacterial ASV composition.

**Factor per analysis**	***df***	***F***	***R2***	***p***
**Overall variation (One-way)**				
Colony size classes	19	1.1520	0.1599	0.027
**Genotype variation (One-way)**				
G1	12	0.9267	0.3079	0.735
G4	11	0.9575	0.3450	0.636
G5	5	1.1300	0.4466	0.288
G6	9	1.1412	0.4829	0.088

Supplementary Figures

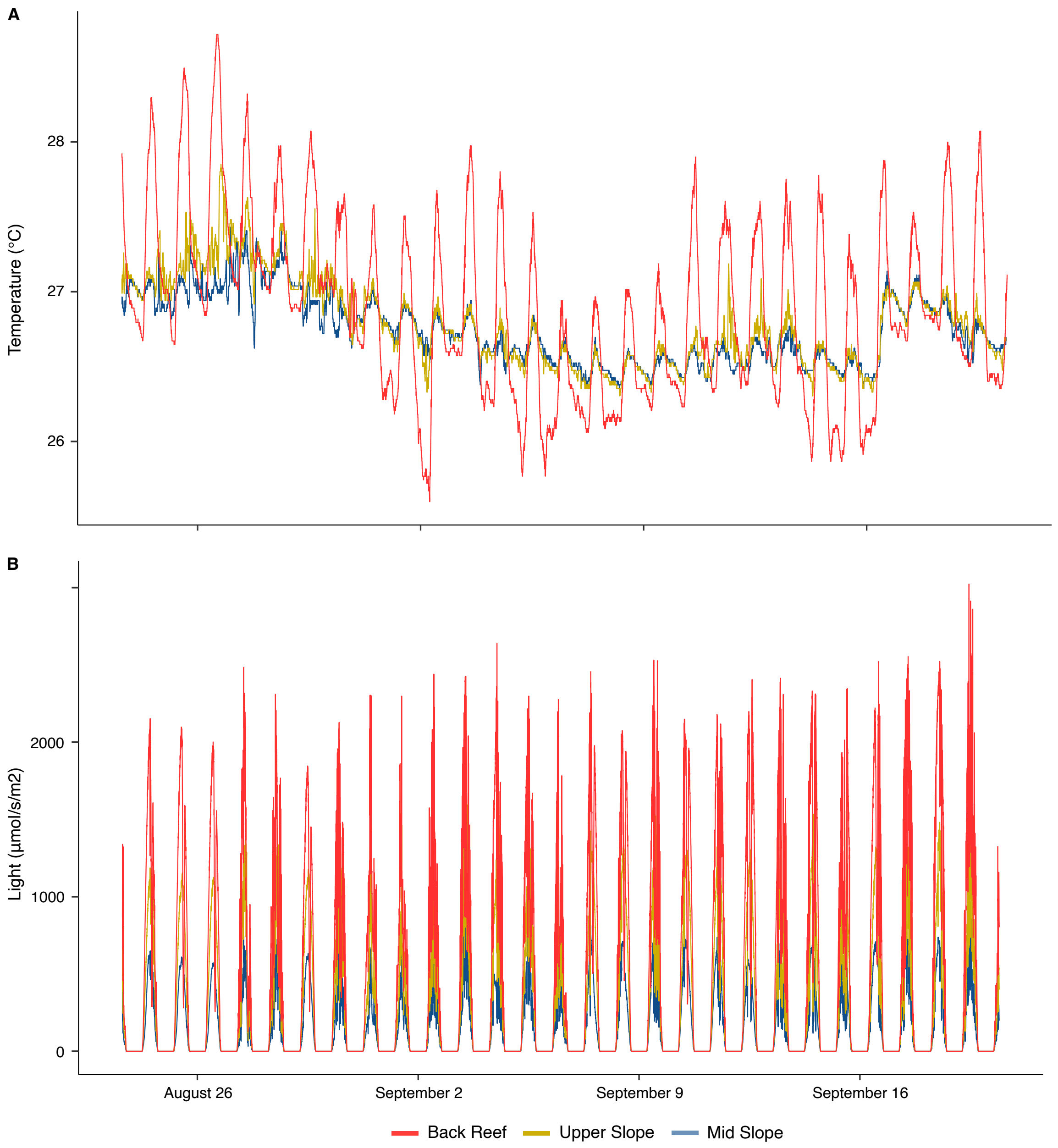

Supplementary Figure 1 | Temperature (**A**) and light (**B**) profiles measured at each of the habitats over time.

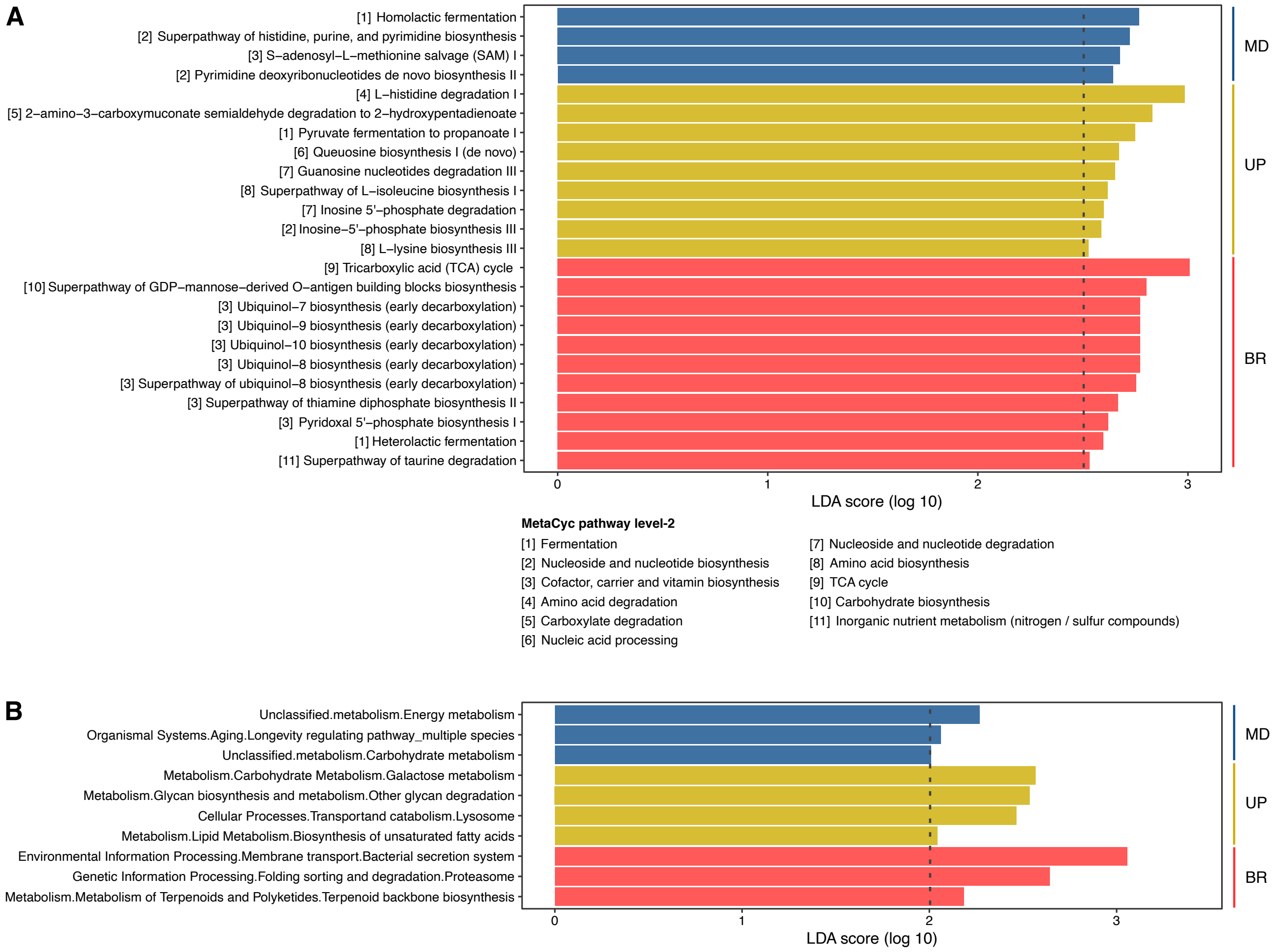

Supplementary Figure 2 | LEfSe analysis displaying predicted functional profiles of the habitat-specific bacterial ASVs. Barplots show the 24 differentially abundant MetaCyc pathways (level 2 in brackets) (**A**) and the 10 KEGG pathways (level 3) (**B**) identified in the microbiomes of particular reef habitats, based on a Linear Discriminant Analysis (LDA, $p < 0.05$ for factorial Kruskal–Wallis and pairwise Wilcoxon tests). Each color denotes the habitat, wherein the distinguishing functional traits were identified (Back reef = red, Upper slope = yellow, Mid slope = blue). Only MetaCyc and KEGG pathways meeting an LDA significant threshold > 2.5 and 2.0, respectively, are shown. The threshold of the logarithmic LDA score is represented by the dotted line. See Supplementary Data 5 for abundances of predicted gene counts of MetaCyc and KEGG pathways (level 1 to 3).
