# Supplementary material for: Naturally occurring fire coral clones demonstrate a genetic and environmental basis of microbiome composition
Source: Nat Commun. 2021 Nov 4;12:6402. doi: 10.1038/s41467-021-26543-x (PMC8568919; doi:10.1038/s41467-021-26543-x)
Supplement: Supplementary file 3 — Description of Additional Supplementary Files [file 41467_2021_26543_MOESM3_ESM.pdf]

Description of Additional Supplementary Information

Naturally occurring fire coral clones demonstrate a genetic and environmental basis of microbiome composition

Dubé CE, Ziegler M, Mercière A, Boissin E, Planes S, Bourmaud CAF, Voolstra CR

Contents

File Name: Supplementary Data 1
Description: Overview over sample colony sizes, geographic coordinates, allelic scores, MLG, genotype, habitat, and morphology IDs**.**

File Name: Supplementary Data 2
Description: Overview over sequence counts, taxonomic classification, and 16S RNA reference amplicon sequence for all 20 144 ASVs.

File Name: Supplementary Data 3
Description: Similarity percentage (SIMPER) analysis of bacterial community composition associated with fire corals (*Millepora* cf. *platyphylla*). Only significant variations of genotype and habitat combinations at the bacterial family level based on one-way PERMANOVA tests and post-hoc tests are shown.

File Name: Supplementary Data 4
Description: IndicSpecies analysis of indicator ASVs/bacterial taxa associated with *Millepora* cf. *platyphylla* genotype and its reef habitat. The association statistic indicates the strength of association for the respective ASV with the tested sample group.

File Name: Supplementary Data 5
Description: Predicted metagenomic traits between genotypes and habitats (per sample data provided). Discriminant traits were determined using linear discriminant analysis (LDA) effect size method with a cut off > 2.5 for each MetaCyc pathway and a cut off > 2.0 for levels 1–3 for individual KOs. No dicriminant traits were identified between genotypes.

File Name: Supplementary Data 6
Description: Overview of weighted Nearest Sequenced Taxon Index (NSTI) scores over all fire coral samples at the genotype and habitat level.
