# Supplementary material for: Naturally occurring fire coral clones demonstrate a genetic and environmental basis of microbiome composition
Source: Nat Commun. 2021 Nov 4;12:6402. doi: 10.1038/s41467-021-26543-x (PMC8568919; doi:10.1038/s41467-021-26543-x)
Supplement: Supplementary file 10 — Reporting Summary [file 41467_2021_26543_MOESM10_ESM.pdf]

nature research

Corresponding author(s): Caroline E. Dubé

Last updated by author(s): Sep 13, 2021

Reporting Summary

Nature Research wishes to improve the reproducibility of the work that we publish. This form provides structure for consistency and transparency in reporting. For further information on Nature Research policies, see our Editorial Policies and the Editorial Policy Checklist.

Statistics

For all statistical analyses, confirm that the following items are present in the figure legend, table legend, main text, or Methods section.

n/a	Confirmed	
☐	☒	The exact sample size (*n*) for each experimental group/condition, given as a discrete number and unit of measurement
☐	☒	A statement on whether measurements were taken from distinct samples or whether the same sample was measured repeatedly
☐	☒	The statistical test(s) used AND whether they are one- or two-sided<br>*Only common tests should be described solely by name; describe more complex techniques in the Methods section.*
☐	☒	A description of all covariates tested
☐	☒	A description of any assumptions or corrections, such as tests of normality and adjustment for multiple comparisons
☐	☒	A full description of the statistical parameters including central tendency (e.g. means) or other basic estimates (e.g. regression coefficient) AND variation (e.g. standard deviation) or associated estimates of uncertainty (e.g. confidence intervals)
☐	☒	For null hypothesis testing, the test statistic (e.g. *F*, *t*, *r*) with confidence intervals, effect sizes, degrees of freedom and *P* value noted<br>*Give P values as exact values whenever suitable.*
☒	☐	For Bayesian analysis, information on the choice of priors and Markov chain Monte Carlo settings
☐	☒	For hierarchical and complex designs, identification of the appropriate level for tests and full reporting of outcomes
☐	☒	Estimates of effect sizes (e.g. Cohen's *d*, Pearson's *r*), indicating how they were calculated

Our web collection on statistics for biologists contains articles on many of the points above.

Software and code

Policy information about availability of computer code

Data collection	The 16S rRNA gene amplicon library was sequenced at the KAUST BioScience Core Laboratory on the Illumina HiSeq 2500 platform using the rapid-run mode with 2 x 250 bp overlapping paired-end reads with a 10 % phiX control. Paired-end sequencing reads were processed with QIIME 2 v2020.6 [1] for quality control, error correction and bacterial taxonomic classification. DADA2 [2] was used for denoising, filtering, merging, and chimera removal from these sequences and generate amplicon sequence variants (ASVs). Sequences were classified taxonomically according to the Silva SSU 138 database [3].<br>[1] Bolyen E. et al. Nat. Biotechnol. 37, 852–857 (2019); [2] Callahan B. et al. Nat. Methods 13, 581–583 (2016); [3] Yilmaz T. et al. Nucl. Acids Res. 42, D643–D648 (2014).
Data analysis	16S rRNA gene-based microbial community analyses were performed in R using different statistical packages: vegan was used for statistical analyses, including similarity percentage (SIMPER) analyses, two- and one-way PERMANOVA tests and Pearson's correlation coefficient [1], ggplot2 for barplots and graphs [2] and IndicSpecies to identify ASVs that were significantly associated with distinct fire coral host genotypes and/or reef habitats [3]. The program Phylogenetic Investigation of Communities by Reconstruction of Unobserved States (PICRUSt2) [4], implemented in the QIIME 2 module called q2-picrust2, was used to predict metagenomic functional content from the 16S rRNA gene. Metagenome predictions were further analyzed using the Galaxy web application (https://huttenhower.sph.harvard.edu/galaxy/) and the LEfSe method [5] to identify significantly different predicted functions of microbial communities among host genotypes and reef habitats.<br>[1] Oksanen J. et al. vegan: Community Ecology Package. (2018); [2] Wickham H. ggplot2: Elegant Graphics for Data Analysis. (Springer, New York, 2016); [3] Cáceres M.D. & Legendre P. Ecology 90, 3566–3574 (2009); [4] Douglas G.M. et al. Nat. Biotechnol. 38, 685–688 (2020); [5] Segata N. et al. Genome Biol. 12, R60 (2011).

For manuscripts utilizing custom algorithms or software that are central to the research but not yet described in published literature, software must be made available to editors and reviewers. We strongly encourage code deposition in a community repository (e.g. GitHub). See the Nature Research guidelines for submitting code & software for further information.

Data

Policy information about availability of data

All manuscripts must include a data availability statement. This statement should provide the following information, where applicable:

- Accession codes, unique identifiers, or web links for publicly available datasets
- A list of figures that have associated raw data
- A description of any restrictions on data availability

The equencing data generated in this study are available under NCBI BioProject ID PRJNA610240 (https://www.ncbi.nlm.nih.gov/bioproject/PRJNA610240). Bacterial ASV reference sequences corresponding to the putative core microbiome are available under GenBank Accession numbers MZ045394–MZ045409 (https://www.ncbi.nlm.nih.gov/nuccore/?term=MZ045394:MZ045409[accn]). Other data generated in this study are available in the Supplementary Data files.

Field-specific reporting

Please select the one below that is the best fit for your research. If you are not sure, read the appropriate sections before making your selection.

☐ Life sciences ☐ Behavioural & social sciences ☒ Ecological, evolutionary & environmental sciences

For a reference copy of the document with all sections, see nature.com/documents/nr-reporting-summary-flat.pdf

Ecological, evolutionary & environmental sciences study design

All studies must disclose on these points even when the disclosure is negative.

Study description

Here we examined bacterial community differences of naturally occurring fire coral clones within and between contrasting reef environments (135 clones, 6 genotypes, 3 reef habitats) to assess the relative contribution of host genotype and environment to microbiome structure. Bacterial community composition of fire coral clones differed between reef habitats, highlighting the contribution of the environment. Similarly, but to a lesser extent, microbiomes varied across different genotypes in identical habitats, denoting the influence of host genotype. The effect and interaction of 'Genotype' and 'Habitat' on bacterial ASV composition was tested using a two-way PERMANOVA, while the effect of 'Genotype' and 'Habitat' was investigated separately using one-way PERMANOVAs. Results of the one-way PERMANOVAs were visualized in non-metric multidimensional scaling (NMDS) ordination plots. For the genetic contribution to variation in bacterial communities, each plot shows one of the three habitats (mid slope, upper slope and back reef) in which the different genotypes were found; overall each genotype had between 13 and 38 replicates: G1 = 38, G2 = 16, G3 = 15, G4 = 32, G5 = 13, G6 = 21. For the environmental contribution to variation in bacterial communities, each plot shows one of the six genotypes with clones found in different habitats; overall each habitat had between 19 to 90 replicates: mid slope = 26, upper slope = 90, back reef = 19. We also found bacterial indicator taxa specific to both, host genotype (irrespective of the environment) and reef habitat (irrespective of clonal identity). Indicator bacterial taxa associated with a given genotype and/or habitat was identified using the IndicSpecies package for corrections of unequal sample sizes with the function Indval.g and only ASVs that were highly significantly ($P < 0.01$) associated with one or several groups were considered: 1-34 genotype-specific ASVs and 3-195 habitat-specific ASVs were identified in this study. At last, our results suggest a functional restructuring of the microbial metabolic network between reef habitats, while the genotype-specific associations seemed to be governed by functional redundancy. Among all functional traits identified using predictive metagenomic analysis, 24 predicted functions distinguished the microbial communities associated with distinct reef habitats (LDA > 2.5), while no discriminant functional traits were identified that differentiated host genotype microbiomes. Taken together, our study argues for a contribution of the microbiome to environmental adaptation, in the form of different taxonomic compositions depending on host genotype.

Research sample

The work presented here is based on a previous collection of nearly 4,000 geo-referenced colonies of fire corals, for which we could demonstrate that clones of the same genotype display different morphologies across variable environments [1]. This work has provided us a unique opportunity to assess the extent of host genetics and environment on microbiome composition by using genetically identical coral hosts across diverse environmental conditions (temperature and light).

Fire corals of the genus Millepora (Cnidaria, Hydrozoa), similar to stony corals (Cnidaria, Scleractinia), are an important component of reef communities worldwide where they shelter symbiotic algae and microbes and build calcareous skeletons, and thus contribute to reef accretion and community dynamics. A recent study of Millepora platyphylla identified several genotypes with clones found across distinct environments on a barrier reef ecosystem in Moorea, French Polynesia [1]. These clones were produced naturally through asexual fragmentation (i.e. likely wave-induced breakage), while dispersed across adjacent habitats (< 210 m apart) via cross-reef transport. Specific environmental gradients across spatially adjacent reef habitats, such as light incidence, temperature, nutrients and water flow (among others), have been reported as underlying factors of substantial variation in the occurrence and persistence of bacterial symbionts. Similar to studying human identical twin microbiome structure and function, fire coral clones provide an ideal natural system to study the relative contribution of host genotype and environment to bacterial association.

For this study, we selected six genotypes with at least four clonal replicates in at least two of the three surveyed habitats, mid slope, upper slope and back reef (n = 135 samples).

[1] Dubé C.E. et al. Mol. Ecol. 26, 3860–3869 (2017).

Sampling strategy

Our sampling design is described in detail in Dubé et al. [1, 2] where fire coral colonies were sampled to investigate the clonal structure and dispersal of sexual propagules between habitats on a barrier reef system. Briefly, between May to September 2013, 3651 fragments of the fire coral M. platyphylla were collected from three adjacent reef habitats located on the north shore of Moorea Island, French Polynesia (17.5267 S, 149.8348 W): the mid slope (13 m depth), upper slope (6 m depth) and back reef (< 1 m depth). Within each habitat, three 300 m-long by 10 m-wide belt transects were laid over the reef, parallel to shore. All colonies of M. platyphylla were geo-referenced by determining their position along the transect-line (0 to 300 m) and straight-line distance from both sides of the transect (0 to 10 m). From these measures, each colony was mapped with x and y coordinates. Small fragments of

tissue-covered skeleton (< 2 cm3) were also collected from each colony using a hammer and chisel, and were preserved in 80 % ethanol for further molecular analysis. All colonies were genotyped using microsatellite markers (as described in [1]) to identify clone mates (i.e., genetically identical colonies produced through asexual fragmentation). From these surveys, 135 colonies were retained to assess the relative contribution from host genotype (6 genotypes) and environment (3 reef habitats) to microbiome structure. The temperature and light intensity were monitored over a one-month period (i.e., from August 23 to September 26, 2019) to assess the environmental differences between the three surveyed reef habitats.
[1] Dubé C.E. et al. Mol. Ecol. 26, 3860–3869 (2017); [2] Dubé C.E. et al. Mol. Ecol. 29, 1508– 1522 (2020).

Data collection	The 16S rRNA gene amplicon library was prepared by C.E.D. and sequenced at the KAUST BioScience Core Laboratory on the Illumina HiSeq 2500 platform using the rapid-run mode with 2 x 250 bp overlapping paired-end reads with a 10 % phiX control. Paired-end sequencing reads were processed by C.E.D. with QIIME2 [1] for quality control, error correction and bacterial taxonomic classification. DADA2 [2] was used for denoising, filtering, merging, and chimera removal from these sequences and generate amplicon sequence variants (ASVs). Sequences were classified taxonomically according to the Silva SSU 138 database [3].<br>[1] Bolyen E. et al. Nat. Biotechnol. 37, 852–857 (2019); [2] Callahan B. et al. Nat. Methods 13, 581–583 (2016); [3] Yilmaz T. et al. Nucl. Acids Res. 42, D643–D648 (2014).
Timing and spatial scale	Field data where collected by C.E.D and A.M. from May to September 2013. DNA extraction and microsatellite genotyping was performed by C.E.D. in 2014, while 16S rRNA gene amplicon library was performed in September 2017 by C.E.D.
Data exclusions	No data were excluded from the analyses.
Reproducibility	PCR amplifications of the 16S rRNA gene were run in triplicate per sample, amplification success was verified on a 1 % agarose gel and successful triplicate reactions were pooled. PCR products were subsequently quantified using a Qubit dsDNA HS Kit (Invitrogen, Carlsbad CA, USA) and run on the Bioanalyzer 2100 (Agilent Technologies, Santa Clara CA, USA) to confirm amplicon length and purity.
Randomization	Randomization was not relevant in our study as we were specifically assessing the bacterial community composition differences in six genotypes of the fire coral Millepora platyphylla with clones occurring naturally in three contrasting reef habitats.
Blinding	Blinding was not relevant in our study as we were specifically assessing the bacterial community composition differences in six genotypes of the fire coral Millepora platyphylla with clones occurring naturally in three contrasting reef habitats.

Did the study involve field work? ☒ Yes ☐ No

Field work, collection and transport

Field conditions	The field work was done by boat when swell surge was low to allow coral sampling using SCUBA and snorkeling at low depth (< 1 m to 13 m). The sampling also occurred in the dry season when rainfall is low.
Location	Fire coral colonies (Millepora platyphylla) were collected from three adjacent reef habitats located at Papetoai on the north shore of Moorea Island, French Polynesia (17.5267 S, 149.8348 W): the mid slope (13 m depth), upper slope (6 m depth) and back reef (< 1 m depth).
Access & import/export	Samples of coral tissue-covered skeleton were exported from Moorea in French Polynesia to Perpignan in France (CITES – FR1298700028–E). Extracted DNA were brought to KAUST, Saudi Arabia.
Disturbance	There were no disturbances as we only collected a small fragment of the coral colony (< 2cm3).

Reporting for specific materials, systems and methods

We require information from authors about some types of materials, experimental systems and methods used in many studies. Here, indicate whether each material, system or method listed is relevant to your study. If you are not sure if a list item applies to your research, read the appropriate section before selecting a response.

Materials & experimental systems

n/a	Involved in the study	
☒	☐	Antibodies
☒	☐	Eukaryotic cell lines
☒	☐	Palaeontology and archaeology
☐	☒	Animals and other organisms
☒	☐	Human research participants
☒	☐	Clinical data
☒	☐	Dual use research of concern

Methods

n/a	Involved in the study	
☒	☐	ChIP-seq
☒	☐	Flow cytometry
☒	☐	MRI-based neuroimaging

Animals and other organisms

Policy information about studies involving animals; ARRIVE guidelines recommended for reporting animal research

Laboratory animals	This study did not involve laboratory animals.
Wild animals	This study involved wild animals, but only a small fragment of coral tissue-covered skeleton was collected in the field.
Field-collected samples	This study involved field-collected samples of corals (only a small fragment).
Ethics oversight	Field experiments were approved by the Presidency of French Polynesia (#0085) and performed in accordance with relevant Polynesian regulations.

Note that full information on the approval of the study protocol must also be provided in the manuscript.
